# Correction: Two-stage matching-adjusted indirect comparison

**DOI:** 10.1186/s12874-022-01753-z

**Published:** 2022-11-01

**Authors:** Antonio Remiro‑Azócar

**Affiliations:** 1 Medical Affairs Statistics, Bayer plc, 400 South Oak Way, Reading, UK; 2 Department of Statistical Science, University College London, 1‑19 Torrington Place, London, UK

**Correction: BMC Medical Research Methodology 22, 217 (2022)**.

10.1186/s12874-022-01692-9

Following publication of the original article [1], the author noticed that two errors were introduced during copyediting.

The equation in “Data-generating mechanisms” should be:

$$y_{i} = \beta_{0} + \boldsymbol{x}_{i}\boldsymbol{\beta}_{\boldsymbol{1}} + \left(\beta_{t} + \boldsymbol{x}_{i}\boldsymbol{\beta}_{\boldsymbol{2}} \right)\mathbbm{1}(t_{i}=1) + \epsilon_{i},$$

The text “Correct specification of theS = 2 covariate distribution” should be “Correct specification of the S = 2 covariate distribution”.

The original article has been updated.
