# Supplementary material for: Two-stage matching-adjusted indirect comparison
Source: BMC Med Res Methodol. 2022 Aug 8;22:217. doi: 10.1186/s12874-022-01692-9 (PMC9358807; doi:10.1186/s12874-022-01692-9)
Supplement: Supplementary file 1 — Additional file 1. Supplementary Material. [file 12874_2022_1692_MOESM1_ESM.pdf]

Supplementary Material of “Two-stage matching-adjusted indirect comparison”

Antonio Remiro-Azócar[1,2]

[1]Medical Affairs Statistics, Bayer Pharmaceuticals
[2]Department of Statistical Science, University College London

Example code

Example `R` code implementing MAIC and 2SMAIC methodologies on a simulated dataset is provided below. The code and data are available at `https://github.com/remiroazocar/Maic2stage` in the `Example` subdirectory. The code is easily adapted to perform the versions of the methods with weight truncation (T-MAIC and T-2SMAIC) by setting `truncation` to `TRUE`. Full code for the simulation study is available in the online repository.

MAIC

```
library("boot") # for non-parametric bootstrap

index.IPD <- read.csv("index_IPD.csv") # load index trial patient-level
    data
comp.ALD <- read.csv("comp_ALD.csv") # load competitor study aggregate-
    level data

set.seed(555) # set seed for reproducibility

# options (truncation==TRUE for T-MAIC)
resamples <- 5000 # number of resamples in non-parametric bootstrap
truncation <- FALSE # whether there is weight truncation
trunc.cutoff <- 0.95 # weights above this percentile are truncated if
    truncation==TRUE

# objective function to be minimized for standard method of moments
Q <- function(alpha, z) {
  return(sum(exp(z %*% alpha)))
}

# function to be bootstrapped
maic.boot <- function(data, indices) {
  dat <- data[indices,] # index trial bootstrap sample
  n <- nrow(dat) # number of subjects in sample
  z <- dat[,c("X1","X2","X3")] # marginal effect modifiers for A vs. C
```

```
  # competitor trial effect modifier means
  theta <- comp.ALD[c("mean.X1", "mean.X2", "mean.X3")]
  k <- ncol(z) # number of effect modifiers
  # center the S=1 effect modifiers on the S=2 means
  z$X1 <- z$X1 - theta$mean.X1
  z$X2 <- z$X2 - theta$mean.X2
  z$X3 <- z$X3 - theta$mean.X3
  # MAIC weight estimation using method of moments
  alpha <- rep(1,k) # arbitrary starting point for the optimizer
  # objective function minimized using BFGS
  Q.min <- optim(fn=Q, z=as.matrix(z), par=alpha, method="BFGS")
  # finite solution is the logistic regression parameters
  hat.alpha <- Q.min$par
  log.hat.w <- rep(0, n)
  for (j in 1:k) {
    log.hat.w <- log.hat.w + hat.alpha[j]*z[,j]
  }
  hat.w <- exp(log.hat.w) # estimated weights
  # weight truncation
  if (truncation==TRUE) {
    # weights above value at specified percentile set to the value at the
       percentile
    w.cutoff <- quantile(hat.w, trunc.cutoff)
    hat.w <- ifelse(hat.w > w.cutoff, w.cutoff, hat.w)
  }
  # fit weighted logistic regression model
  outcome.fit <- glm(y~trt, weights=hat.w, data=dat)
  # fitted treatment coefficient is marginal effect for A vs. C
  hat.Delta.10 <- coef(outcome.fit)["trt"]
  return(hat.Delta.10)
}

# ordinary non-parametric bootstrap with replacement
boot.object <- boot::boot(data=index.IPD, statistic=maic.boot, R=resamples)
# bootstrap mean of marginal A vs. C treatment effect estimate
hat.Delta.10 <- mean(boot.object$t)
# bootstrap variance of A vs. C treatment effect estimate
hat.var.Delta.10 <- var(boot.object$t)
# published B vs. C marginal treatment effect
hat.Delta.20 <- with(comp.ALD, hat.Delta)
# reported variance for B vs. C marginal treatment effect
hat.var.Delta.20 <- with(comp.ALD, var.hat.Delta)
# indirect treatment comparison (A vs. B)
hat.Delta.12 <- hat.Delta.10 - hat.Delta.20
hat.var.Delta.12 <- hat.var.Delta.10 + hat.var.Delta.20
# construct Wald-type confidence intervals based on normal distributions
uci.Delta.12 <- hat.Delta.12 + qnorm(0.975)*sqrt(hat.var.Delta.12)
lci.Delta.12 <- hat.Delta.12 + qnorm(0.025)*sqrt(hat.var.Delta.12)
```

2SMAIC

```
library("boot") # for non-parametric bootstrap

index.IPD <- read.csv("index_IPD.csv") # load index trial patient-level
    data
comp.ALD <- read.csv("comp_ALD.csv") # load competitor study aggregate-
    level data

set.seed(555) # set seed for reproducibility

# options (truncation==TRUE for T-2SMAIC)
resamples <- 5000 # number of resamples in non-parametric bootstrap
truncation <- FALSE # whether there is weight truncation
trunc.cutoff <- 0.95 # weights above this percentile are truncated if
    truncation==TRUE

# objective function to be minimized for standard method of moments
Q <- function(alpha, z) {
  return(sum(exp(z %*% alpha)))
}

# function to be bootstrapped
maic2s.boot <- function(data, indices) {
  dat <- data[indices,] # index trial bootstrap sample
  n <- nrow(dat) # number of subjects in sample
  ### trial assignment model ###
  z <- dat[,c("X1","X2","X3")] # marginal effect modifiers for A vs. C
  # competitor trial effect modifier means
  theta <- comp.ALD[c("mean.X1", "mean.X2", "mean.X3")]
  k <- ncol(z) # number of effect modifiers
  # center the S=1 effect modifiers on the S=2 competitor means
  z$X1 <- z$X1 - theta$mean.X1
  z$X2 <- z$X2 - theta$mean.X2
  z$X3 <- z$X3 - theta$mean.X3
  # MAIC (trial assignment) weight estimation using method of moments
  alpha <- rep(1,k) # arbitrary starting point for the optimizer
  # objective function minimized using BFGS
  Q.min <- optim(fn=Q, z=as.matrix(z), par=alpha, method="BFGS")
  # finite solution is the logistic regression parameters
  hat.alpha <- Q.min$par
  log.hat.w <- rep(0, n)
  for (j in 1:k) {
    log.hat.w <- log.hat.w + hat.alpha[j]*z[,j]
  }
  hat.w <- exp(log.hat.w) # estimated trial assignment weights
  ### treatment assignment model ###
  trt.model <- glm(trt~X1+X2+X3, data=dat, family=binomial(link="logit"))
  # predict propensity scores (probability of assignment to actual
    treatment)
  dat$hat.e <- predict(trt.model, data=dat, type="response")
  # inverse probability of treatment weights
  hat.iptw <- ifelse(dat$trt==1, 1/dat$hat.e, 1/(1-dat$hat.e))
  # combine weights
  hat.omega <- hat.w*hat.iptw
```

```
  # weight truncation
  if (truncation==TRUE) {
    # weights above value at specified percentile set to the value at the
        percentile
    omega.cutoff <- quantile(hat.omega, trunc.cutoff)
    hat.omega <- ifelse(hat.omega > omega.cutoff, omega.cutoff, hat.omega)
  }
  # fit weighted logistic regression model
  outcome.fit <- glm(y~trt, weights=hat.omega, data=dat)
  # fitted treatment coefficient is marginal effect for A vs. C
  hat.Delta.10 <- coef(outcome.fit)["trt"]
  return(hat.Delta.10)
}

# ordinary non-parametric bootstrap with replacement
boot.object <- boot::boot(data=index.IPD, statistic=maic2s.boot, R=
    resamples)
# bootstrap mean of marginal A vs. C treatment effect estimate
hat.Delta.10 <- mean(boot.object$t)
# bootstrap variance of A vs. C treatment effect estimate
hat.var.Delta.10 <- var(boot.object$t)
# published B vs. C marginal treatment effect
hat.Delta.20 <- with(comp.ALD, hat.Delta)
# reported variance for B vs. C marginal treatment effect
hat.var.Delta.20 <- with(comp.ALD, var.hat.Delta)
# indirect treatment comparison (A vs. B)
hat.Delta.12 <- hat.Delta.10 - hat.Delta.20
hat.var.Delta.12 <- hat.var.Delta.10 + hat.var.Delta.20
# construct Wald-type confidence intervals based on normal distributions
uci.Delta.12 <- hat.Delta.12 + qnorm(0.975)*sqrt(hat.var.Delta.12)
lci.Delta.12 <- hat.Delta.12 + qnorm(0.025)*sqrt(hat.var.Delta.12)
```
